# Spatially Conditioned Speech Timing: Evidence and Implications

**DOI:** 10.3389/fpsyg.2019.02726

**Published:** 2019-12-05

**Authors:** Jason A. Shaw, Wei-rong Chen

**Affiliations:** ^1^Department of Linguistics, Yale University, New Haven, CT, United States; ^2^Haskins Laboratories, New Haven, CT, United States

**Keywords:** feedforward control, articulatory phonology, gesture coordination, CV timing, Mandarin Chinese, electromagnetic articulography, state-based feedback, neutral attractor

## Abstract

Patterns of relative timing between consonants and vowels appear to be conditioned in part by phonological structure, such as syllables, a finding captured naturally by the two-level feedforward model of Articulatory Phonology (AP). In AP, phonological form – gestures and the coordination relations between them – receive an invariant description at the inter-gestural level. The inter-articulator level actuates gestures, receiving activation from the inter-gestural level and resolving competing demands on articulators. Within this architecture, the inter-gestural level is blind to the location of articulators in space. A key prediction is that intergestural timing is stable across variation in the spatial position of articulators. We tested this prediction by conducting an Electromagnetic Articulography (EMA) study of Mandarin speakers producing CV monosyllables, consisting of labial consonants and back vowels in isolation. Across observed variation in the spatial position of the tongue body before each syllable, we investigated whether inter-gestural timing between the lips, for the consonant, and the tongue body, for the vowel, remained stable, as is predicted by feedforward control, or whether timing varied with the spatial position of the tongue at the onset of movement. Results indicated a correlation between the initial position of the tongue gesture for the vowel and C-V timing, indicating that inter-gestural timing is sensitive to the position of the articulators, possibly relying on somatosensory feedback. Implications of these results and possible accounts within the Articulatory Phonology framework are discussed.

## Introduction

Patterns of relative timing between consonants and vowels appear to be conditioned in part by abstract phonological structure, such as syllables, but also modulated by the particular gestures being coordinated (e.g., Marin and Pouplier, 2010; Marin, 2013; Brunner et al., 2014; Shaw and Gafos, 2015; Hermes et al., 2017; Ying et al., 2017). The most rigorous attempts to formalize phonologically relevant temporal patterns have come within the Articulatory Phonology (AP) framework, which draws a distinction between the inter-gestural level of representation and the inter-articulator level (Browman and Goldstein, 1989; Saltzman and Munhall, 1989). In AP, context-independent phonological representations are given at the inter-gestural level, in the form of dynamical systems that exert task-specific forces on articulators. The form of the dynamical system for a gesture remains constant across different phonological and lexical contexts. Contextual effects on articulatory behavior, due to the starting position of the articulators or to temporal co-activation of gestures, is resolved at the inter-articulator level. The same gesture can have different net effects on articulatory behavior in different contexts owing to the way that competing demands on an articulator are resolved at the inter-articulator level. Crucially, AP is a feedforward control system. Gestures (at the inter-gestural level) exert forces on articulators but do not receive feedback from the state of the articulators in space or time. Feedback of this sort is encapsulated within the inter-articulator level.

The two-level feedforward control system of AP accounts for some language-specific phonetic patterns. It can account for target undershoot phenomenon and context effects on articulation without sacrificing phonological constancy (Browman and Goldstein, 1990). Moreover, higher level phonological structures have been linked to characteristic patterns of timing between gestures, results which receive a natural account within the inter-gestural level of AP. For example, languages that allow syllables with complex onsets, such as English, Polish and Georgian, pattern together in how word-initial consonant clusters are coordinated to the exclusion of languages that disallow complex onsets, such as Arabic and Berber (Goldstein et al., 2007; Shaw and Gafos, 2015; Hermes et al., 2017). In addition to simplex vs. complex syllables onsets, segment complexity may also have a temporal basis (Shaw et al., 2019). Shaw et al. (2019) show that in palatalized stops of Russian, e.g., /p^j^/, the labial and lingual gestures are timed synchronously whereas superficially similar sequences in English, e.g., /pj/in/pju/“pew”, and unambiguous sequences in Russian, e.g.,  /br/, are timed sequentially. This difference between complex segments and segment sequences mirrors behavior found at the syllabic level. Language-specific temporal organization of phonology, as illustrated by cases such as these receives a natural account within the inter-gestural level of AP.

In contrast to AP, neuro-anatomical models of speech production rely on auditory and somatosensory state feedback to control movement timing (Houde and Nagarajan, 2011; Hickok, 2014). In these models there are no context-independent dynamics comparable to the gestures of AP. Rather, articulation is controlled through the mechanism of feedback. Adjustments to articulation are made online in order to guide articulators to producing target sounds. While these models are silent on the phonological phenomena for which the inter-gestural level of AP provides a natural explanation, they provide an account for how some speakers adjust articulation online in response to perturbation of auditory feedback (e.g., Houde and Jordan, 1998). In AP, articulator position information is available only to the inter-articulator level, which is governed by the Task Dynamics model (Saltzman and Munhall, 1989). Within the inter-articulator level, Task Dynamics assumes perfect information about articulator positions, although more recent work has explored replacing this assumption with a more realistic model of feedback (Ramanarayanan et al., 2016). Crucially for our purposes, there is no mechanism for state-based feedback at the inter-articulator level to influence inter-gestural coordination. This means that while auditory/somatosensory feedback could drive articulatory adjustments to how a particular task is achieved it cannot trigger earlier/later activation of a gesture.

Experimental evidence indicating that information from the articulator level can feed back to the inter-gestural level is available from perturbation studies. In experimental contexts when there is a physical perturbation to articulation, gestures have been observed to “reset” (Saltzman, 1998; Saltzman et al., 1998). Phase-resetting in response to physical perturbation suggests that coordination at the inter-gestural level does not uni-directionally drive articulatory movement. Saltzman et al. (1998) argue: “intergestural and interarticulatory dynamics must be coupled bidirectionally, so that feedback information can influence the intergestural clock in a manner that is sensitive to articulatory state (p. 422).”

Some recent kinematic studies suggest possible links between the spatial position of articulators and relative timing observable outside of perturbation experiments (Brunner et al., 2014; Pastätter and Pouplier, 2017). Brunner et al. (2014) list the spatial position of the articulator as one of a number of factors that influences measures of gesture coordination, leading to consonant-specific variation in timing patterns in German. Pastätter and Pouplier (2017) investigated whether coarticulatory resistance, a measure of the degree to which an articulator resists spatial perturbation (Bladon and Al-Bamerni, 1976; Recasens and Espinosa, 2009; Chen et al., 2015) influences the relative timing of a consonant and following vowel. In line with their hypotheses, overlap between a consonant and vowel was affected by the coarticulatory resistance of the consonant. C-V overlap was greater for consonants less resistant to coarticulation. Pastätter and Pouplier also report a corresponding effect of consonant identity on the spatial position of the vowel. Vowels that showed less temporal overlap with the preceeding consonant were spatially closer to the preceeding consonant, converging evidence that consonants with high coarticulatory resistance delay vowel movements. In order to account for this pattern, Pastätter and Pouplier proposed to vary coupling strength at the intergestural level by articulator. In this way, different articulators could enter into the same basic coordination relation, e.g., in-phase or anti-phase timing, but exert differential forces on vowel timing. The theoretical account offered by Pastätter and Pouplier makes properties of articulators (but not their spatial positions) visible to inter-gestural timing. The account preserves language-specific timing at the inter-gestural level and feedforward control but does not reconcile the need for state-based feedback observed by Saltzman et al. (1998).

Our aim in this paper is to provide a direct test of whether the spatial position of the tongue influences consonant-vowel (C-V) coordination. To do so, we conducted an Electromagnetic Articulography (EMA) study of Mandarin Chinese. Mandarin is a good language to investigate C-V coordination, both because of its phonological properties and because it is relatively well-studied otherwise. Mandarin allows fairly free combination of tones with consonants and vowels to make CV monosyllabic words. Varying lexical tone, while keeping the consonant and vowel sequence constant allowed us to generate a comparatively large number of phonologically distinct monosyllables to test our research question. We focused on non-low back vowels in Mandarin because past work has shown that variation in lexical tone for these vowels does not influence the spatial location of the vowel target; /i/ and /a/, in contrast, vary with tone (Shaw et al., 2016). Our stimuli were CV monosyllables, consisting of a labial consonant and a back vowel. Single-syllable words in isolation allow for considerable variability in the starting position of the articulators. Across the observed variation in the spatial position of the tongue body, we investigated whether inter-gestural coordination between the lips, for the consonant, and the tongue body, for the vowel, remained constant, as is predicted by feedforward control.

There are competing hypotheses about the feedforward control regime for Mandarin C-V syllables. Xu (2005) theorizes that consonants and vowels (as well as lexical tones) begin synchronously, at the start of the syllable. This assumption has been implemented in computational modeling of *f*_0_ for tone and intonation (Xu and Wang, 2009; Xu et al., 2015). A slightly different conclusion about Mandarin CV timing was reached by Gao (2008, 2009). In an EMA experiment tracking tongue and lip movements, Gao (2009) found that there is positive C-V lag, i.e., the vowel gesture does not begin movement until after the onset of movement of the consonant. Gao attributed the positive C-V lag to competitive coordination between consonant, vowel, and tone gestures. The account incorporates pressure to start the consonant and vowel at the same time, i.e., in-phase coordination, along with other competing demands on coordination. The tone and vowel are coordinated in-phase, but the consonant (C) and tone (T) are coordinated sequentially (anti-phase). The competing demands of anti-phase C-T timing, in-phase C-V, and in-phase C-T timing are resolved by starting the vowel at the midpoint between the onset of consonant and tone gestures. Notably, Gao’s analysis of C-V lag in Mandarin mirrors the analysis of C-V timing in languages with syllable-initial consonant clusters (Browman and Goldstein, 2000; Gafos, 2002; Goldstein et al., 2007; Marin and Pouplier, 2010; Hermes et al., 2013, 2017; Marin, 2013; Shaw and Gafos, 2015). The common thread is that the observed C-V lag in a CCV syllable is driven by competing forces on inter-gestural coordination – anti-phase coordination for the consonants and in-phase coordination between each onset consonant and the vowel. Xu et al. (2015) do not address Gao’s data. However, both accounts of C-V lag in Mandarin described above, although they differ in assumptions, involve feed-forward control of articulation. As such, they predict that relative timing is blind to the spatial position of the articulator. In the experiment that follows, we test this hypothesis.

## Experiment

### Speakers

Six native speakers of Mandarin Chinese (3 male) participated. They were aged between 21 and 25 years (*M* = 23.7; SD = 1.5) at the time of the study. All were born in Northern China (Beijing and surrounding areas) and lived there until at least 18 years of age. The speakers all lived in Sydney, Australia, where the experiment was conducted, at the time of their participation. All participants were screened by a native speaker of Mandarin Chinese to ensure that they spoke standard Mandarin. Procedures were explained to participants in Mandarin by the second author, a speaker of Taiwanese Mandarin. Participants were compensated for their time and local travel expenses.

### Materials

Target items were a set of CV monosyllables that crossed all four lexical tones of Mandarin, tone 1 “high”, tone 2 “rise”, tone 3 “low”, and tone 4 “fall” with two labial consonants {/m/, /p/} and three back rounded vowels {/ou/, /u/, /uo/} yielding 24 items, which were repeated 6–12 times by each speaker producing a corpus of 949 tokens for analysis. We chose labial consonants because of the relative independence between the consonant (lips) and the vowel (tongue dorsum) gestures. We chose back vowels in particular because of past work showing that /u/ in Mandarin resists the coarticulatory effects of tone, which influence /i/ and /a/ (Shaw et al., 2016). We also report an analysis of unrounded /i/ and /a/, drawing on data from Shaw et al. (2016). The purpose of this additional analysis is to assess whether the pattern for our target items generalizes to unrounded vowels.

Target items were randomized with fillers and displayed one at a time on a monitor in Pinyin, a standard Romanization of Chinese. The three back vowels included in the materials have the following representation in Pinyin: “o” /uo/, “u” /u/, “ou” /ou/. Here and throughout, we use slashes to refer to IPA symbols. Orthographic representations of vowels not in slashes refer to Pinyin. Many of the items were real words and could have been displayed as Chinese characters. We chose to represent the items with Pinyin orthography because it allowed us to collect all combinations of the onset consonants, vowels and tones under study including those that do not correspond to real words. The Pinyin sequences that are not attested words were combinations of /p/ with /ou/.

### Equipment

We used an NDI Wave Electromagnetic Articulograph system sampling at 100 Hz to capture articulatory movement. We attached sensors to the tongue tip (TT), body (TB), dorsum (TD), upper lip (UL), lower lip (LL), lower incisor (Jaw), nasion and left/right mastoids. Acoustic data were recorded simultaneously at 22 KHz with a Schoeps MK 41S supercardioid microphone (with Schoeps CMC 6 Ug power module).

### Stimulus Display

Syllables were displayed in Pinyin on a monitor positioned outside of the NDI Wave magnetic field 45 cm from participants. Stimulus display was controlled manually using a visual basic script in Excel. This allowed for online monitoring of hesitations, mispronunciations and disfluencies. These were rare, but when they occurred, participants were asked to repeat syllables.

### Post-processing

**FIGURE 1:**
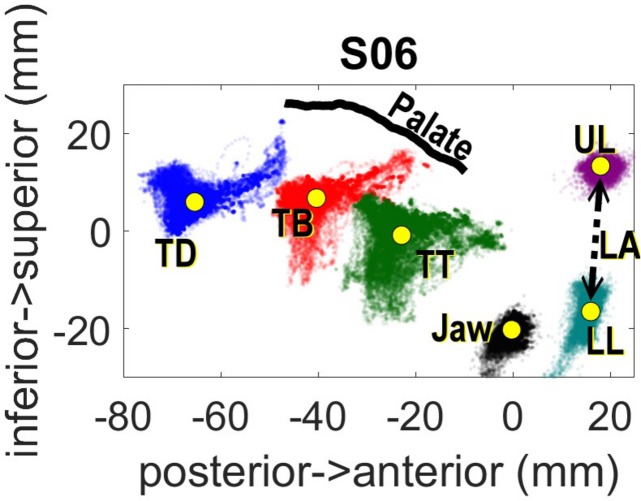
Spatial distribution of EMA sensors across the experiment for one subject.

Head movements were corrected computationally after data collection with reference to the left/right mastoid and nasion sensors. The post-processed data was rotated so that the origin of the spatial coordinates is aligned to the occlusal plane. The occlusal plane was determined by having each participant hold between their teeth a rigid object (plastic protractor) with three sensors configured in a triangle shape. Lip Aperture (LA), defined as the Euclidean distance between the upper and lower lip sensors, was also computed following rotation and translation to the occlusal plane. Figure 1 shows the range of movement for the entire experiment for one speaker following head correction.

### Articulatory Analysis

The articulatory data analysis focuses on the relative timing between consonant and vowel gestures, which we define in terms of temporal lag, and the position of EMA sensors at linguistically relevant spatio-temporal landmarks: the *onset* of articulatory movement and the achievement of the gestural *target*. Onset and target landmarks were determined according to thresholds of peak velocity in the movement trajectories. For the labial consonants, the Lip Aperture trajectory was used. For the back vowels, landmarks were determined with reference to the Tongue Dorsum sensor in the anterior-posterior dimension (i.e., TDx). Landmark labeling was done using the *findgest* algorithm in MVIEW, a program developed by Mark Tiede at Haskins Laboratories (Tiede, 2005).

**FIGURE 2:**
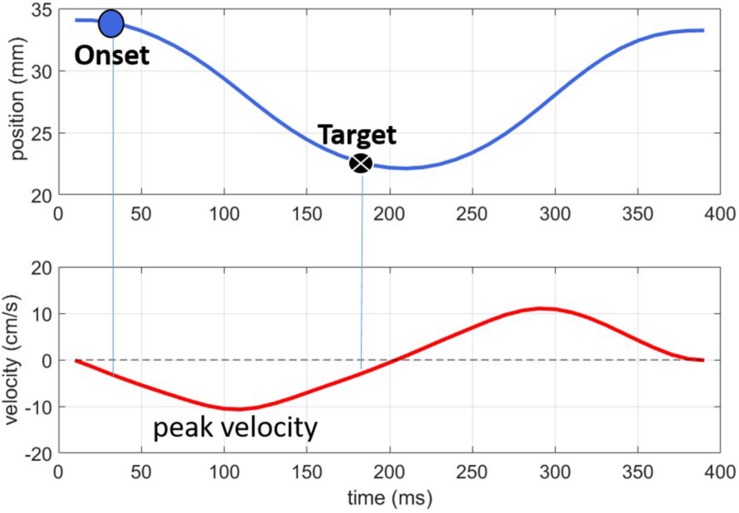
Illustration of the onset and target landmarks for a labial consonant. The **top panel** shows lip aperture over time; the **bottom panel** shows the corresponding velocity signal.

Figure 2 shows an example of how the articulatory landmarks, labeled on the Lip Aperture signal (top panel) relate to the velocity peaks (lower panel). As the lips move together for the labial consonant, the lip aperture (top panel) gradually narrows. The peak velocity in this closing phase of −10 cm/s occurs just after 100 ms. The signal was thresholded at 20% of this velocity peak, resulting in the Onset and Target landmarks. We also explored the velocity minimum as a possible articulatory landmark for analysis but found that the threshold of peak velocity provided more reliable measurements across tokens. The cause seemed to be that some of the monophthongs in the experiment tended to have relatively long periods of low velocity around the point of maximum opening corresponding to the vowels. Although the NDI Wave system produced high spatial resolution recordings, even a small degree of measurement error (∼0.6 mm) makes picking out the true velocity minima from the wide basin of low velocity movement subject to sizeable temporal variation. Using the threshold of peak velocity mitigates the effect of measurement noise, providing a reliable vowel target landmark across tokens.

**FIGURE 3:**
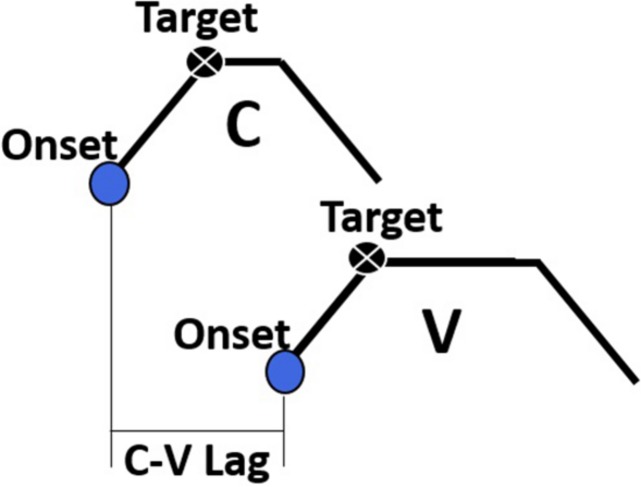
A schematic depiction of the C-V lag measurement, the interval between the onset of the consonant gesture and the onset of the vowel gesture.

The primary dependent variable of interest in this study was the temporal lag between consonants and vowels, henceforth C-V lag. A schematic diagram of C-V lag is provided in Figure 3. C-V lag was determined by subtracting the timestamp of the gesture onset of the consonant, ${\text{C}}_{\text{ts}}^{\text{onset}}$, from the timestamp of the gesture onset of the vowel, ${\text{V}}_{\text{ts}}^{\text{onset}}$:

$$\text{CVlag}={\text{V}}_{\text{ts}}^{\text{onset}}-{\text{C}}_{\text{ts}}^{\text{onset}}$$

The primary independent variable of interest is the distance between the tongue at movement onset for the vowel and at the achievement of target. We quantified this in a few different ways. First, we measured the spatial position of the TD sensor at the onset of movement of the vowel. Since all of the target vowels in this study were back vowels, the primary movements for the vowels involved tongue retraction, i.e., movement from a more anterior position to a more posterior position. We refer to the position of the tongue dorsum in this dimension as TDx:

TDx=coordinate⁢of⁢the⁢tongue⁢dorsum⁢sensor⁢in⁢theanterior⁢-⁢posterior⁢dimension

For the speaker shown in Figure 1, the range of TDx values is about 18 mm, i.e., from −42 to −60 mm. The negative coordinates are relative to the occlusal plane, so −60 mm indicates 60 mm behind the occlusal plane clenched in the participants’ teeth. The value of TDx at movement onset for the vowel served as the key independent measure in the study. The closer the value of TDx at vowel onset was to zero, the further the tongue would have to move to achieve its target.

In addition to TDx at movement onset, we also measured more directly how far away the tongue was from its target at the onset of movement. We call this measure *Tdist*, for distance to target. We used inferior-superior (*y*) and anterior- posterior (*x*) dimensions for both TD and TB in the calculation. Hence, Tdist is the four-dimensional Euclidean distance between the position of lingual sensors (TB, TD) at the onset of vowel movement and at the vowel target. The vowel target for each subject was determined by averaging the position of these sensors at the *target* landmark across tokens of the vowel. The formula for Tdist is defined below:

Tdist=

$$\sqrt{\begin{array}{c} {\left({\text{TD}}_{\text{x}}^{\text{Onset}}-\text{mean}\,\left({\text{TD}}_{\text{x}}^{\text{Target}}\right)\right)}^{2}+{\left({\text{TD}}_{\text{y}}^{\text{Onset}}-\text{mean}\,\left({\text{TD}}_{\text{y}}^{\text{Target}}\right)\right)}^{2}\\ +{\left({\text{TB}}_{\text{x}}^{\text{Onset}}-\text{mean}\,\left({\text{TB}}_{\text{x}}^{\text{Target}}\right)\right)}^{2}+{\left({\text{TB}}_{\text{y}}^{\text{Onset}}-\text{mean}\,\left({\text{TB}}_{\text{y}}^{\text{Target}}\right)\right)}^{2} \end{array}}$$

**FIGURE 4:**
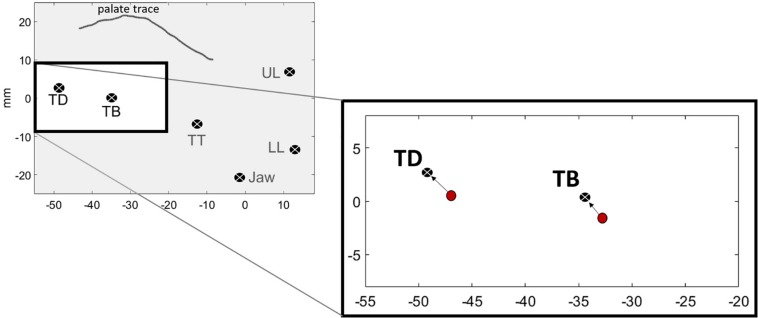
Vowel targets for /uo/ for one speaker, calculated as the average position of the TD and TB sensors across repetitions. Red circles show the spatial positions of the sensors at the onset of movement toward the vowel target. The black circles with the white “x” denote the vowel target. The arrows represent the Euclidean distance between the sensors at the onset of movement and the achievement of target.

Figure 4 shows a visual representation of Tdist. The left panel shows the average position of the sensors for one speaker’s “o” /uo/ vowel. The right panel shows the TB and TD components of Tdist as directional vectors in 2D (*x,y*) space. The start of the vector is the position of the sensors at the onset of movement, represented as red circles. The end of the vectors are the vowel targets for TB and TD. The length of the arrow from the vowel onset to the vowel target is the Euclidean distance for each sensor. Tdist is the combination of the two vectors.

Our main analysis assesses the effect of TDx and Tdist on C-V lag. To do this, we fit a series of nested linear mixed effects models to C-V lag. All models contained a random intercept for subject. We explored a baseline model with fixed effects for VOWEL (o, u, ou), CONSONANT (b, m), and TONE (1, 2, 3, 4). We ultimately dropped TONE from the baseline model because it did not improve over a model with just VOWEL and CONSONANT as fixed effects. This was somewhat expected since we deliberately selected vowels unlikely to be influenced by tone. Both remaining fixed factors in the baseline model were treatment coded – “o” /uo/ was the reference category for VOWEL and “b” /p/ was the reference category for CONSONANT. To this baseline model, we added one of our main factors of interest: TDx or Tdist. We also investigated whether another kinematic variable, peak velocity of the vowel gesture, explained C-V lag above and beyond the variables related to TD position at the onset of movement, i.e., TDx and Tdist. The modeling results are given in the next section following some visualization and description of the main factors of interest.

## Results

### Effect of Spatial Position on C-V Lag

**FIGURE 5:**
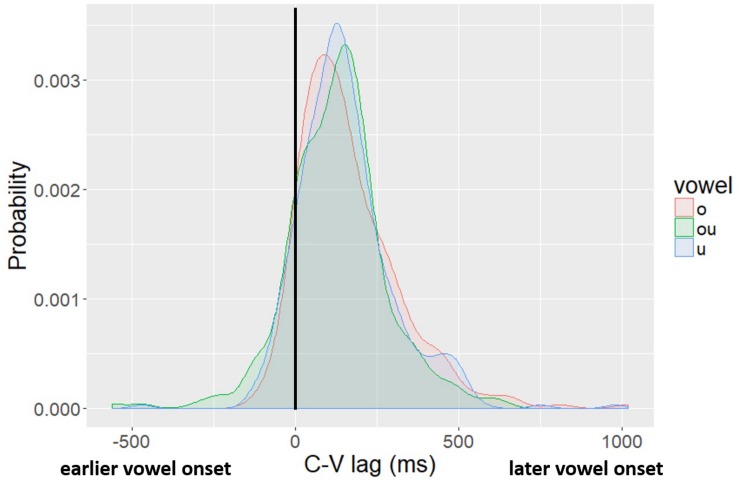
Kernel density plot of lag values by vowel. The legend shows the Pinyin for the vowels, which correspond to: “o” /uo/, “ou” /ou/, “u” /u/.

Figure 5 shows the probability density functions of C-V lag in raw milliseconds (i.e., not normalized) for the three vowels, fitted by kernel density estimations. We report the distribution in milliseconds to facilitate comparison across studies. The solid black vertical line at the 0 point indicates no lag – the vowel and the consonant start at the same time. In tokens with negative lag (the left side of the figure) the vowel started movement before the consonant; in tokens with a positive lag (right side of the figure), the consonant starts movement before the vowel. The distribution of lag values is centered on a positive lag for all three vowels, indicating that, on average, vowel movement follows consonant movement. Moreover, the size of the lag is comparable to what has been reported in past studies of CV lag in Mandarin (Gao, 2009; Zhang et al., 2019) and other lexical tone languages (Karlin and Tilsen, 2015; Hu, 2016; Karlin, 2018). There is also, however, substantial variation. The main aim of this paper is to evaluate whether the variability observed in CV lag is related to variability in the spatial position of the tongue dorsum at the onset of movement.

**FIGURE 6:**
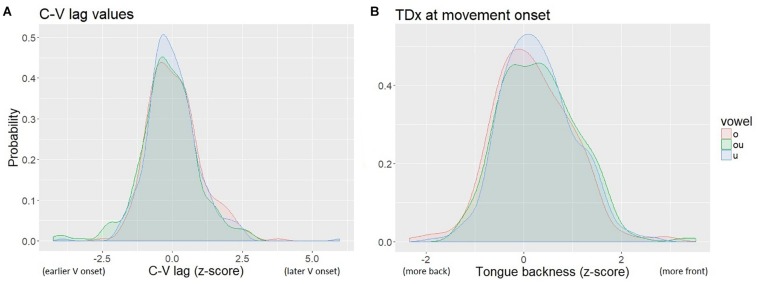
Kernal density plot of normalized C-V lag **(A)** and TDx **(B)**. The legend shows the Pinyin for the vowels, which correspond to: “o” /uo/, “ou” /ou/, “u” /u/.

The distribution of tongue backness values (as indicated by TDx at the onset of movement of the TD toward the vowel target) was multi-modal, due to inter-speaker variation in the size of the tongue and the placement of the TD sensor. To normalize for speaker-specific sensor location and lingual anatomy, we calculated *z*-scores of TDx within speaker. The normalized values are centered on 0. We also normalized the C-V lag measures by *z*-score. The normalized measures of C-V lag and TDx are shown in Figure 6. The resulting distributions for both TDx and C-V lag are roughly normal.

**FIGURE 7:**
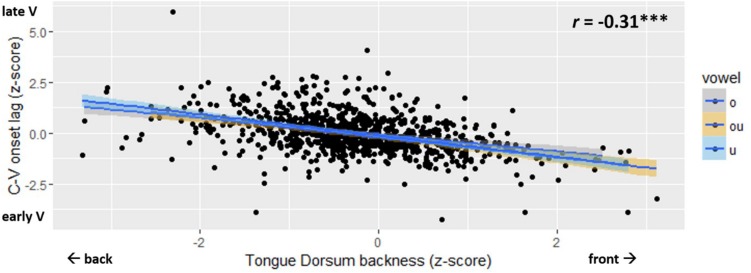
Scatter plot of C-V lag (*y*-axis) and Tongue Dorsum backness (*x*-axis). The legend shows the Pinyin for the vowels, which correspond to: “o” /uo/, “ou” /ou/, “u” /u/.

The main result is shown in Figure 7. The normalized measure of C-V lag is plotted against TDx, i.e., tongue dorsum backness at movement onset. The figure shows a significant negative correlation (*r* = −0.31; *p* < 0.001). Variation in C-V lag is correlated with variation in the spatial position of the tongue dorsum at the onset of movement. C-V lag tends to be shorter when the tongue dorsum is in a more anterior position at movement onset. When the starting position of the TD is more posterior, i.e., closer to the vowel target, C-V lag is longer. Thus, Figure 7 shows that the vowel gesture starts earlier, relative to the consonant gesture, when it has farther to go to reach the target. To evaluate the statistical significance of the correlation in Figure 7, we fit linear mixed effects models to C-V lag, using the lme4 package (Bates et al., 2014) in R. The baseline model included a random intercept for speaker and fixed effects for vowel quality and onset consonant. A second model added the main fixed factor to the baseline model. To index the position of the tongue dorsum relative to the vowel target, we considered both TDx and Tdist as fixed factors. For both of these factors as well as for C-V lag, we used the z-score-normalized values in all models. The normalized values of TDx and Tdist were highly collinear (*r* = 0.48^∗∗∗^), which prevents us from including both in the same model.

As expected, the effects of these factors on C-V lag were quite similar. The correlation between Tdist and C-V lag was slightly weaker (*r* = −0.28^∗∗∗^) than the correlation between TDx and C-V lag. Adding TDx to the model led to a slightly better improvement over baseline than Tdist. We therefore proceed by using TDx as our primary index of the starting position of the tongue dorsum.

We also considered whether the speed of the vowel movement impacts C-V lag. The peak velocity of articulator movements is known to be linearly related to gesture magnitude, i.e., the displacement of the articulator in space (Munhall et al., 1985; Ostry and Munhall, 1985). For this reason, TDx, which, as shown above, is strongly correlated to Tdist, is also highly correlated with the peak velocity of the movement (*r* = 0.33, *p* < 0.001). The natural correlation between peak velocity and displacement can be normalized by taking the ratio of peak velocity to displacement, a measure sometimes referred to as kinematic stiffness (Adams et al., 1993; Shaiman et al., 1997; Perkell et al., 2002; Van Lieshout et al., 2007). This provides a kinematic measure of speed that can be assessed across variation in TDx. We evaluated the correlation between stiffness and C-V lag and found that there was no effect (*r* = −0.03). This indicates that gesture velocity, once gesture magnitude is factored in, has no effect of C-V lag.

Adding TDx resulted in significant improvement to the baseline model (χ^2^ = 125.52; *p* < 2.20E-16). Moreover, the increased complexity of the model is justified by the variance explained. The six degrees of freedom in the baseline model increased to seven degrees of freedom in the baseline + TDx model, but the AIC and BIC scores were lower in the baseline + TDx model (AIC_baseline_ = 2607.2, AIC_baseline+TDx_ = 2483.7; BIC_baseline_ = 2636.3, BIC_baseline+TDx_ = 2517.7). This indicates that the spatial position of the tongue dorsum has a significant effect on inter-gestural timing.

A summary of the fixed effects for our best model, baseline + TDx, is as follows. VOWEL had only a marginal effect on C-V lag. The effect of CONSONANT was negative (β = −0.276; *t* = −4.722^∗∗∗^), indicating that syllables that begin with [m] have shorter C-V lag than those that begin with [p], the intercept category for the consonant factor. The strongest fixed factor in the model was that of TDx (β = −0.559; *t* = −12.245^∗∗∗^). The strong negative effect indicates, as shown in Figure 7, that C-V lag decreases with increases in TDx. Larger TDx values indicate a more anterior position of the tongue. Since the vowel targets in the stimuli were all posterior (back vowels), the negative effect of TDx can be interpreted as shorter C-V lag values in tokens with more front starting positions for the vowel. In other words, the farther the tongue dorsum is from the (back) vowel target, the earlier the movement starts (and, thus, the shorter the C-V lag).

### Exemplification of the Main Result

**FIGURE 8:**
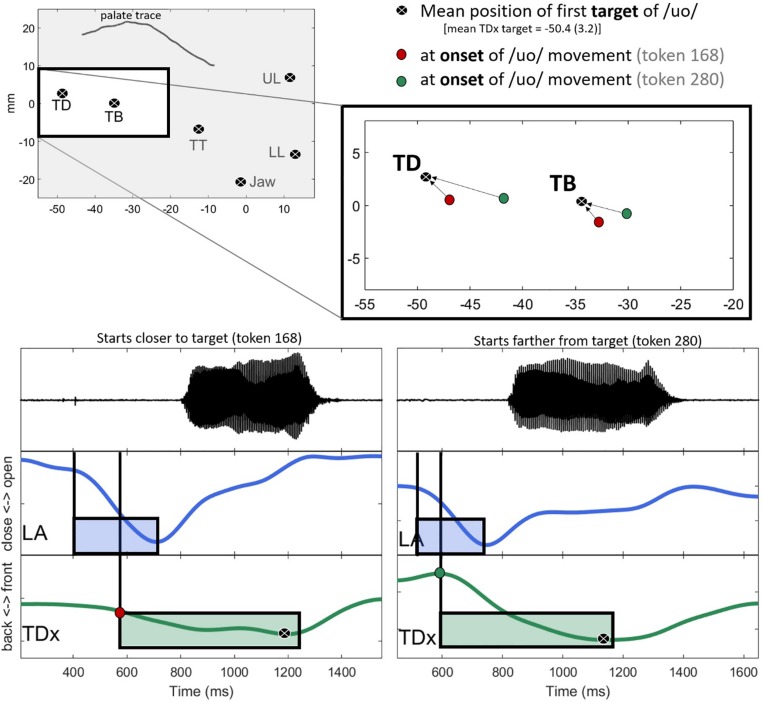
Comparison of two tokens differing in both the backness of the tongue dorsum (TDx) at the onset of vowel movement and C-V lag. In line with the general trend in the data, C-V lag is greater when the tongue dorsum is closer to the target **(left)** than when it is further away **(right)**.

The general trend in the data is that C-V lag decreases with the anteriority of the tongue. To put this another way, movement toward the vowel target (relative to the consonant) is delayed when the tongue happens to be already near the target position. This pattern is exemplified with specific tokens in Figure 8. The top left panel shows the mean position of the sensors at the target of /uo/ for one speaker. At the target, the average backness of the TD sensor is −50.4(3.2) mm (black circles). The panel on the upper right zooms in on the position of the TB and TD sensors for two tokens, token 168, shown as red circles is relatively close to the vowel target for /uo/. Token 280, in contrast, is further away (green circles). The bottom two panels compare the time course of movement for each of these tokens. The panel on the left shows token 168, which starts closer to the target. In line with the general trend in the data, movement toward the target in token 168 is somewhat late relative to the lip aperture gesture. TD movement toward the target does not start until about halfway through the closing phase of the labial gesture. The TD movement in token 280, shown on the right, starts earlier in the phase of the consonant. Consequently, the lag between the consonant gesture and the vowel gesture is shorter in token 280 (right) than in token 168 (left).

### Extension to Unrounded Vowels

The target items in this study involved labial consonants followed by rounded vowels. As described above, we selected high back vowels since they are known to resist tonal coarticulation. However, since high back vowels in Mandarin Chinese are rounded, there is a potential for interaction between gestural control of the lips by the labial consonant and gestural control by the rounded vowel. While the particular nature of this interaction for Mandarin is not known, some possibilities include gestural blending, whereby the movement of the lips results from a compromise between temporally overlapped task goals, or gesture suppression, whereby one of the overlapping gestures takes full control of the articulator. In the task dynamics model, these outcomes are dictated by the blending strength parameter (Saltzman and Munhall, 1989), which is hypothesized to be language specific (Iskarous et al., 2012). In some languages, the labial and dorsal components of high back rounded vowels enter into a trading relation such that the degree of rounding, for, e.g., /u/, varies with the degree of tongue dorsum retraction (Perkell et al., 1993). This raises the question – to what extent is our main result related to the presence of rounding for the vowels? To address this question, we extended our analysis to unrounded vowels, /a/ and /i/, drawing on EMA data reported in Shaw et al. (2016).

**FIGURE 9:**
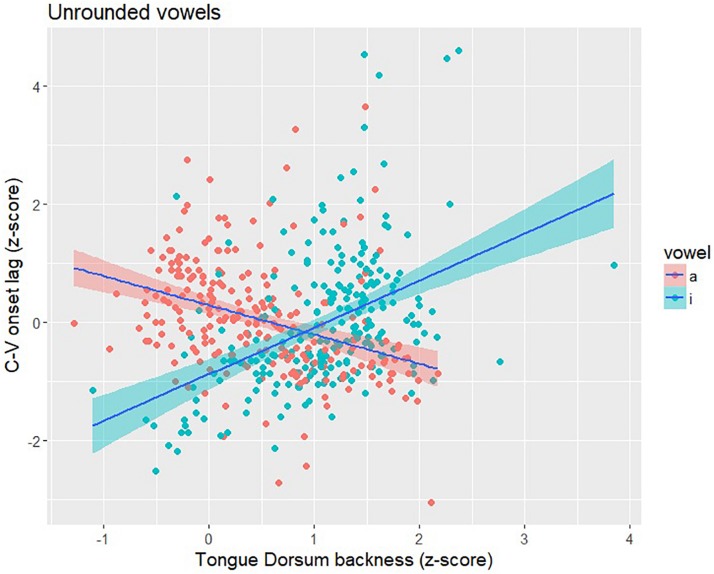
Scatter plot of C-V lag (*y*-axis), as indexed by the onset of the gestures, and Tongue Dorsum backness (*x*-axis), as indexed by TDx at the onset of movement, for /pa/ and /pi/ syllables. Larger values of TDx indicate a more front tongue position. For /pa/, there is a negative correlation – shorter C-V lag when TD is more front (farther from the /a/ target) and longer C-V lag when TD is more back (closer to the /i/ target). For /pi/, there is a positive correlation – longer C-V lag when the TD is more back (farther from the /i/ target) and longer C-V lag when TD is more front (closer to the /i/ target).

The items in Shaw et al. (2016) included multiple repetitions of /pa/ and /pi/ produced with all four Mandarin tones by the same six speakers analyzed in this study. Following the procedure outlined in section “Experiment”, we calculated C-V lag and TDx position for /pa/ and /pi/ syllables. A total of 470 tokens (233 /pa/ tokens; 237 /pi/ tokens) were analyzed. Both syllables show a correlation between C-V lag and TDx that is similar in strength to what we observed for high back vowels (Figure 7). For /pa/, the direction of the correlation was negative (*r* = −0.36; *p* < 0.001), the same direction as for the high back vowels. When the tongue dorsum is in a more front position (farther from the /a/ target), C-V lag tends to be shorter, indicating an earlier vowel movement relative to the consonant; when the tongue dorsum is in a more back position (closer to the /a/ target), C-V lag is longer. We observed the same pattern for the low back vowel, which is unrounded, as we observed for the high back vowels, which are rounded. The correlation between C-V lag and TDx is similarly strong for /pi/ syllables (*r* = 0.45; *p* < 0.001), but the correlation is positive. The positive correlation for /pi/ makes sense given the anterior location of the vowel target. In contrast to the back vowels, a relatively front tongue dorsum position puts the tongue close to the /i/ target; in this case, C-V lag tends to be long, indicating a delayed vowel gesture onset (relative to the consonant). Figure 9 provides a scatterplot of C-V lag and TDx for /pi/ and /pa/. The positive correlation for /pi/ is essentially the same pattern as the negative correlation observed for /pa/ and for the high back vowels that served as the main target items for the study. From this we conclude that whatever the effect of vowel rounding is on the lip gestures in Mandarin, it does not seem to have any influence on the relation between TDx position at the onset of the vowel gesture and C-V lag. We observe the same pattern across rounded and unrounded vowels.

## Discussion

Analysis of C-V lag in Mandarin monosyllables confirmed patterns reported in the literature and also revealed new effects that have theoretical implications for models of speech timing control.

First, we found that C-V lag in the Mandarin syllables in our corpus, which all have lexical tone, tends to be positive. The vowel typically starts well after the consonant. This pattern, positive C-V lag, has been reported for Mandarin before (Gao, 2008, 2009) and for other lexical tone languages (Karlin and Tilsen, 2015; Hu, 2016; Karlin, 2018). C-V lag tends to be longer for languages with lexical tone than for languages that have intonational tones or pitch accents (Mücke et al., 2009; Niemann et al., 2011; Hermes et al., 2012). In terms of millisecond duration, the C-V lag in tone languages reported in the studies above is in the range of ∼50 ms while the C-V lag for languages that lack lexical tone tends to be smaller, ∼10 ms. The C-V lag in our study was substantially longer (roughly twice) than other reports of lexical tone languages (Figure 5). This difference in absolute duration is probably due at least in part to the nature of our stimuli. Monosyllables read in isolation in Pinyin encourages hyperarticulation but served the specific purpose in our study of allowing variation in tongue position at the onset of movement while controlling for other factors that could influence C-V timing in longer speech samples. Another possible reason for the longer absolute C-V lag in our materials could be the onset consonants. Studies of tone and intonation tend to select sonorant consonants as stimuli to facilitate continuous tracking of   *f*_0_ across consonants and vowels. Our stimuli included both a nasal onset consonant, /m/, and an oral onset consonant, /p/. Although this was not expected, there was a significant effect of onset consonant identity on C-V lag. C-V lag was significantly shorter in syllables beginning with the nasal stop than in syllables beginning with the oral stop. The longer C-V lag found in our materials overall is conditioned in part by our inclusion of oral plosive onsets. As to why oral plosives condition longer C-V lag (than nasals), we currently have no explanation.

We found no effect of tone on C-V lag and only a negligible effect of vowel. Syllables with all four Mandarin tones and all three back vowels showed similarly positive C-V lag. The lack of a tone effect was expected from past work on Mandarin, including Gao (2008). We avoided /i/ and /a/ vowels in our target items because past research had shown that the target tongue position for these vowels varies across tones whereas /u/ has a stable target (Shaw et al., 2016). Conceivably, the effect of tone on C-V lag would be more complicated for other vowels, because a change in tone may also condition a change in the magnitude of tongue displacement toward the vowel target. The vowel written with Pinyin “o” after labial consonants is pronounced as a diphthong /uo/ in standard Mandarin; the first target of this diphthong is the same target as for the monophthong /u/. The third vowel in the study was /ou/, which is also in the high back space. From the standpoint of feed-forward models of timing, effects of vowel quality on C-V coordination are not expected in general. This study does not offer a particularly stringent test of this assumption, since the vowel targets were similar. Rather, the materials in this study were optimized to evaluate effects of variation at the onset of the vowel.

We found a significant effect of the main factor of interest in this study. The spatial position of the tongue dorsum at the onset of vowel movement had a significant effect on C-V lag. We also showed that this main pattern generalized to /a/ and /i/ by re-analyzing data from Shaw et al. (2016). C-V lag values showed substantial token-by-token variation (Figure 5); however, the variation was not random. Variation in when the vowel movement starts relative to the consonant was systematically related to the spatial position of the tongue dorsum. When the tongue dorsum was further forward – farther from the vowel target – movement started earlier than when the tongue dorsum was further back – closer to the vowel target. This type of behavior is not expected from a strictly feedforward model of relative timing control, such as the coupled oscillator model of inter-gestural timing (Goldstein and Pouplier, 2014). However, the results are not inexplicable. There are a range of possible explanations. Before moving on to discuss possible theoretical explanations for the pattern, we first address a potential limitation of the study.

Our strategy of eliciting words in isolation was successful in that we obtained variation in the starting position of the tongue dorsum. The structure of this variation played an important role in revealing the main result. Since the stimuli consisted of labial consonants followed by vowels, each trial ended with the mouth in an open position (for production of the vowel) and the next trial began with a labial gesture, requiring either narrowing of the lips (/f/ in some filler trials) or closure (/m/, /p/). This design allows for the possibility that participants take up a rest posture in between trials which involves lip closure. In labeling the gestures for further analysis, we noticed that the lips typically remained open until the onset of the labial gesture; however, a small number of tokens involved lip closures that were unusually early, possibly because the lips closed before active control associated with the target stimuli. These tokens show up as outliers to the statistical distribution for the lip aperture gesture, i.e., extra long closure duration. Since our analysis did not exclude statistical outliers, we consider here the possible impact that they could have on our main result.

To assess the role of outliers resulting from early closure, we re-ran our analysis excluding outliers using each of two well-established methods: *a priori* trimming and outlier removal through model critique (Baayen and Milin, 2015). The mean lip aperture duration in the data was 327 ms (SD = 117); the median was 300 ms (27 ms shorter than the mean), which, consistent with our token-by-token observations from labeling, suggests a skew toward longer duration outliers. Following the *a priori* trimming method, we excluded tokens from analysis that were three standard deviations from the mean lip aperture duration value and re-fit the nested lmer models reported above. Removing outliers in this way improved the model fit, as indicated by a lower AIC:2382 for trimmed data set, c.f., 2483 for full data set. The effect of TDx on C-V lag was reduced slightly following *a priori* trimming, as indicated by the coefficient estimate for TDx: for the trimmed data set β = −0.53(SE = 0.043), c.f., for the full data set β = −0.56 (SE = 0.046). The slight change in the coefficient is reflected as well in the pearson’s correlation between C-V lag and TDx: *r* = −0.30 for the trimmed data set vs. *r* = −0.31 for the full data set. We also removed outliers via model critique. Following the method suggested in Baayen and Milin (2015), we removed outliers to our best fitting model. Residuals to model fit greater than three standard deviations were removed and the model was refit to the trimmed data set. The resulting model showed further improvement; AIC dropped to 2297. The coefficient for TDx decreased slightly β = −0.52 (SE = 0.043). The pearson’s correlation between C-V lag and TDx was the same as for the a prior trimming: *r* = −0.30. Removing outliers based on model fit does not directly reference lip aperture duration. Nevertheless, this approach produced similar results to removing outliers with unusually long lip closure duration (*a priori* trimming). Removing outliers based on lip closure duration had the effect of improving model performance overall with only a negligible influence on the estimate for TDx. This suggests that the occasional long labial closure in the data introduced noise (unexplained variance) in the model but did not have a substantial influence on the observed relation between spatial position (TDx) and intergestural timing (C-V lag).

We focus the remainder of this discussion on two possible explanations for the main result (section “Downstream Targets” and “Neutral Attractors”) as well as some additional theoretical implications (section “Additional Theoretical Implications”).

### Downstream Targets

**FIGURE 10:**
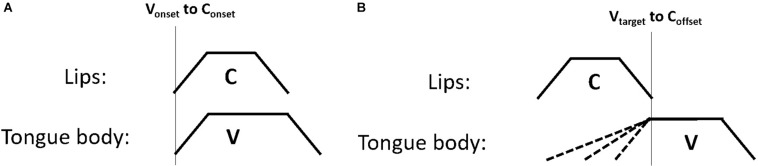
Two schematic diagrams of timing relations. Panel **(A)** shows the onset of the vowel timed to the onset of the consonant; panel **(B)** shows the target of the vowel timed to the offset of the consonant.

One possible explanation is that gesture coordination makes use of a richer set of gestural landmarks than just gesture onsets. For example, Gafos (2002) proposes a set of five articulatory landmarks which are referenced by a grammar of gestural coordination. These landmarks include the onset of movement, the achievement of target, the midpoint of the gesture plateau (or “c-center”), the release from target and the offset of controlled movement (p. 271). Variation in gesture onsets, as we observed for the vowel movements in this study could potentially subserve later production goals, such as the coordination of the target landmark or others landmarks that occur later in the unfolding of the gesture, i.e., after the gesture onset. To illustrate this concept, Figure 10 shows two coordination schemas. The left panel, Figure 10A shows a pattern of synchronous consonant and vowel gestures. In this schema the vowel onset is aligned to the consonant onset – the two gestures are in-phase. This can be contrasted with Figure 10B, which shows a later vowel target. The target of the vowel in this case is timed to the offset of the consonant gesture. The coordination schema dictates that the vowel achieves its spatial target at the offset of controlled movement for the consonant. If the coordination relation controlling C-V timing references the vowel target (and not the vowel onset), the vowel onset would be constrained only by the requirement that the target is achieved at the end of the consonant gesture. This could dictate that the timing of the vowel onset varies as a function of its distance to the vowel target. This account suggests some degree of state-feedback from articulator position to inter-gestural timing control. If the onset of the vowel gesture is timed to achieve its target at the end of the consonant gesture, speech motor control must have access to the position of the tongue, i.e., state feedback, either through proprioception or through tactile information.

To assess the downstream target hypothesis we calculated the lag between the vowel target and two other landmarks in the consonant gesture, the consonant *release* and consonant *offset*. These two landmarks were defined according to thresholds of peak velocity in the movement away from the consonant constriction, i.e., the positive velocity peak in Figure 2. Accordingly, they are the release-phase equivalents of the onset and target landmarks.

**FIGURE 11:**
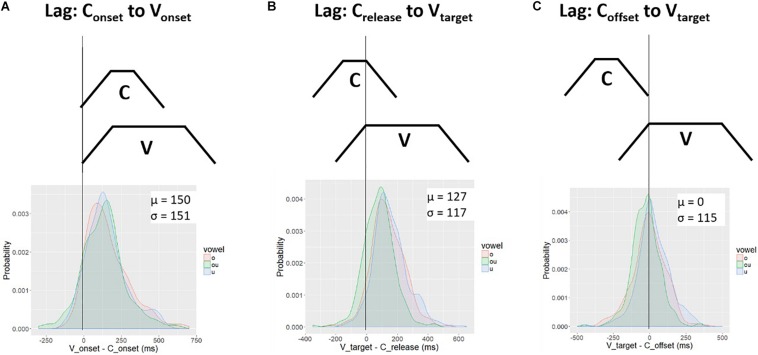
Temporal lag between three sets of C-V landmarks: **(A)** C_onset_ to V_onset_; **(B)** C_release_ to V_target_; **(C)** C_offset_ to V_target_. The top row shows a schema for the lag measurement. The schema represents the C-V timing pattern under which the lag measure is zero (perfect alignment). The bottom row shows the distribution of lag values. Lag measures were computed by subtracting the vowel landmark from the consonant landmark. The average lag between the C_offset_ and V_target_ **(C)** is zero; in contrast, the average lag for the schemas in **(A)** and **(B)** is positive.

Figure 11 shows the distribution of lag values for C_release_ to V_target_ (Figure 11B) and for C_offset_ to V_target_ (Figure 11C). These are obtained by subtracting the consonant landmark from the vowel landmark, V_target_ - C_offset_. For comparison, the lag values for C_onset_ to V_onset_, first presented in Figure 5, are repeated as Figure 11A. The top panels show schemas of lag measurements and the bottom panels show kernel density plots. In each plot a vertical black line is drawn at the 0 point. For C_onset_ to V_onset_ (Figure 11A) and C_release_ to V_target_ (Figure 11B), the lag is positive (on average). For C_offset_ to V_target_ (Figure 11C), the probability mass is centered on zero. Although there is substantial variability around the mean, the target of the vowel occurs, on average, at the offset of the consonant. This pattern is consistent with the downstream target hypothesis. The target of the vowel is aligned to the offset of the consonant. In order to achieve the vowel target at the offset of consonant movement, movement toward the vowel target must start during the consonant gesture. How much earlier in time the vowel gesture starts is free to vary with the spatial position of the relevant articulators.

The alignment between C_offset_ and V_target_ (Figure 11C) has a possible alternative explanation. Since the vowels of our target items are rounded, it is possible that C_offset_ corresponds to an articulatory landmark associated with the labial component of the vowel instead of the consonant release phase. A hint of this possibility is apparent in the lip aperture (LA) signal in Figure 8 (left), token 168, which shows a multi-stage time function. There is an abrupt decrease in LA velocity at around 900 ms; after this change, LA widens more slowly until around 1200 ms, when the TD achieves its target. It is possible that control of LA passes smoothly from the consonant gesture to a vowel gesture in such a way that the threshold of peak velocity applied to LA picks up on the labial component of the vowel, instead of the actual C_offset_, which could occur earlier, i.e., around 900 ms in token 168. We therefore pursue another set of predictions that can differentiate the alignment schemas in Figure 10.

To further evaluate the alignment schemas in Figure 10, we conducted an analysis that leverages the temporal variability in the data. Articulatory coordination, like biological systems more generally, exhibit variation, owing to a wide range of factors. In assessing the predictions of control structures, such as the coordination schema in Figure 10B, we therefore look to the patterns of variability that are uniquely predicted. This approach follows past work exposing coordination relations by examining how they structure temporal variability in kinematic (Shaw et al., 2009, 2011; Gafos et al., 2014; Shaw and Gafos, 2015).

**FIGURE 12:**
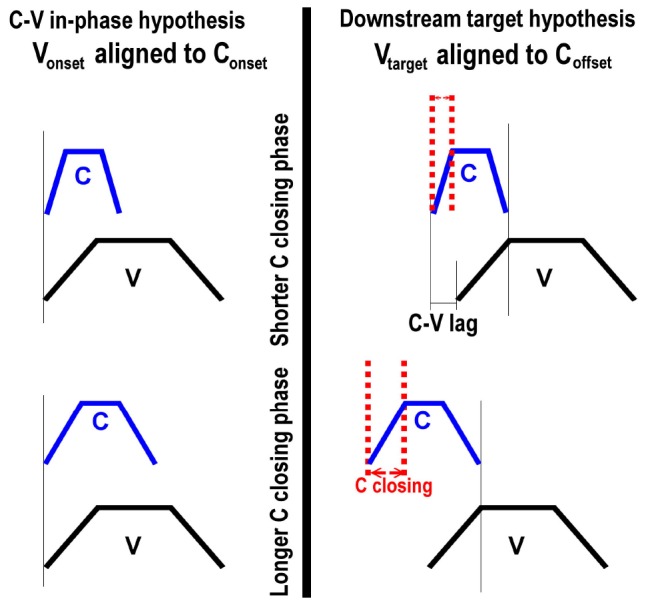
Comparison of two C-V coordination schema under different consonant durations. The **top panels** show shorter consonants and the **bottom panels** show longer consonants. As consonant duration increases from the **top panel** to the **bottom panel**, C-V lag is increased only for the schema on the right, where the vowel target is timed to the release of the consonant.

To exemplify, consider Figure 12. The top panels repeat the schema in Figure 10; the bottom panels show the same schema with longer consonant gestures. As the consonant gesture increases in length from the top panels to the bottom panels, we observe different effects on C-V lag. In the left panel, where the vowel onset is timed to the consonant onset, there is no effect of consonant duration on C-V lag. In the right panel, in contrast, C-V lag increases with consonant duration. Since the vowel is timed to the offset of the consonant, a longer consonant entails longer C-V lag (assuming that gesture duration for the vowel remains constant). This prediction can also be tested in our data. Moreover, testing this prediction does not require that we disentangle the release of the labial consonant from the labial component of the vowels. If the vowel target is timed to any landmark of the consonant following the consonant target, then an increase in consonant duration predicts an increase in C-V lag.

**FIGURE 13:**
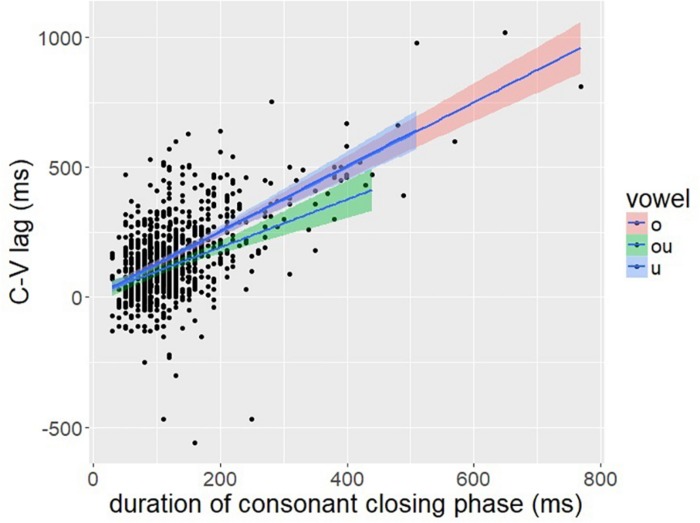
A scatter plot of C-V lag (*y*-axis) and the duration of the closing phase of the consonantal gesture.

To evaluate this prediction, we investigated the correlation between C-V lag and the closing phase of the consonant. The closing phase of the consonant was defined as the duration from the onset of consonant movement to the achievement of target in the lip aperture signal, defined by a threshold of peak velocity (see Figure 2). A positive correlation between C-V lag and consonant duration is predicted by the downstream target hypothesis (Figure 12: right) but not by the C-V in-phase hypothesis (Figure 12: left). If the consonant and vowel gestures are in-phase, then C-V lag should be unaffected by consonant duration. The correlation between C-V lag and consonant duration was quite high (*r* = 0.61, *p* < 0.001), which is consistent with the downstream target prediction. A scatter plot is shown in Figure 13.

Figure 13 shows that temporal variation in C-V lag is structured in a manner consistent with Figure 12: right. Variation in consonant duration stems from numerous factors, including individual differences that may have a neuro-muscular basis (Crystal and House, 1988; Tsao and Weismer, 1997; Tsao et al., 2006). Nevertheless, this variability is useful in exposing the underlying control structure. As consonant duration varies, C-V lag also varies in a manner predicted by downstream targets, as in Figure 10B, but not by in-phase timing, Figure 10A. The significant correlation is predicted by any alignment pattern in which the vowel target is timed to a consonant landmark later than the consonant target. Despite variation in speech rate and the absolute duration of consonantal and vocalic intervals, we observe consistency in temporal covaration predicted by a specific pattern of gesture coordination. Shaw and Gafos (2015) report a similar result for English. The pattern of temporal variation found across 96 speakers followed the predictions of a common pattern of gestural coordination, even as the absolute duration of consonant and vowel intervals varied substantially.

While our discussion has focused so far on intergestural timing, i.e., the timing of the vowel gesture relative to the consonant, the target-based timing account described above also suggests something about intra-gestural control that can be tested in the data. The vowel gesture may start earlier in time when it has farther to go to reach the target and starts later in time when there is less distance to travel. Stated this way, the timing of the vowel onset is relative not to the consonant (i.e., inter-gestural timing) but to the distance to the vowel target, i.e., gesture amplitude. Notably, this particular relation is one that is predicted by a non-linear dynamical system with an anharmonic potential and not by a linear dynamical system (Sorensen and Gafos, 2016: 204).

**FIGURE 14:**
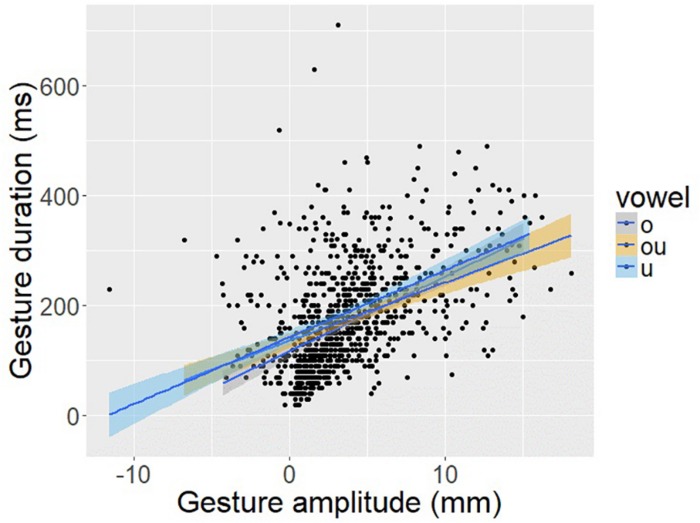
Scatter plot of vowel gesture duration (*y*-axis), as measured from the onset of movement to the achievement of target based on the TDx trajectory, and gesture amplitude (*x*-axis) as measured from the degree of TD sensor displacement in the anterior-posterior dimension (i.e., TDx).

To provide a direct test of this hypothesis about intra-gestural timing, Figure 14 plots vowel gesture amplitude, as indexed by the displacement of TDx from vowel onset to vowel target, against the duration of the opening phase of the vowel, as indexed by the temporal interval from vowel onset to vowel target. There is a significant positive correlation between gesture amplitude and gesture duration (*r* = 0.45; *p* < 0.001). This result helps to sharpen the interpretation of the C-V lag results as well. It appears that the vowel gesture starts earlier when it has farther to go to reach the target, an aspect of intra-gestural control consistent with a non-linear dynamical systems model of the gesture.

We were curious as well about whether the variation in vowel gesture onset has consequences for acoustic vowel duration. Since the onset of vowel gestures typically takes place sometime during the consonant closure, variation in the gesture onset is potentially masked in the acoustics by the overlapped consonant. To investigate this, we measured the interval from the acoustic onset of the vowel, as indicated by the onset of formant structure, to the articulatory vowel target (as per Figure 2). This acoustic interval of the vowel was *not* positively correlated with the magnitude of the vowel gesture (TDx). There was a slight negative correlation (*r* = −0.15, n. s.). This indicates that the strong correlation between gesture magnitude and gesture duration is largely masked in the acoustic vowel interval from onset of voicing to the vowel target. The distance of the tongue to the vowel target (gesture amplitude), which is significantly correlated with vowel start times and is reflected in C-V lag, does not correlate with acoustic vowel duration.

### Neutral Attractors

A second possible explanation for the main result is that there is a neutral attractor at work. Neutral attractors have been hypothesized to take control of articulators that are not otherwise under gesture control (Saltzman and Munhall, 1989). When a gesture achieves its target, control of the model articulator falls to the neutral gesture, which will drive the articulator toward a neutral position.

The explanation of the main result – that TD position correlates with C-V lag – in terms of a neutral attractor is as follows. Consider again two tokens that differ in the position of the TD during the pre-speech period of silence (Figure 8). When the TD is at an extreme position, the neutral attractor drives it toward a neutral position before the vowel gesture takes control. The momentum of the articulator movement controlled by the neutral attractor carries over to gestural control by a vowel. On this account, vowels with more extreme tongue dorsum positions may appear to start earlier in time relative to the overlapped consonant because control of the TD passes smoothly from a neutral attractor to a vowel gesture. In contrast, when the TD is already in a neutral position, movement does not start until the vowel gesture is activated. On this account, the early onset of vowel gestures that begin far from targets is an epiphenomenon of neutral attractor control.

**FIGURE 15:**
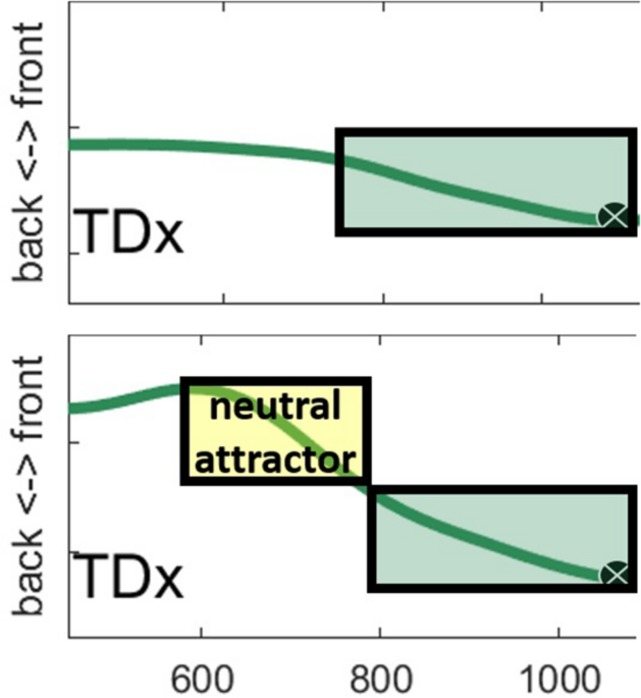
Two vowel tokens from our EMA data are shown with hypothetical gesture control structures overlaid. The **top panel** illustrates a case in which only a vowel gesture controls movement. The **bottom panel** illustrates a case in which a neutral attractor first brings the TD from an extreme front position to a less extreme position before the vowel gesture takes control.

The contrast between a token with early TD movement and one with later movement is shown in Figure 15. The top panel shows the token with a non-extreme TD backness position. The green box shows the vowel gesture activation interval, terminating with the achievement of target. The bottom panel illustrates the neutral attractor proposal. The yellow box shows the neutral attractor which drives the TD away from an extreme front position. Since the vowel target is back, the neutral attractor happens to be driving the TD in the same direction as the vowel gesture, which kicks in at the same time across tokens. Typical heuristics for parsing gesture onsets from EMA trajectories based on the velocity signal, including those used in this paper, would likely be unable to differentiate between movement associated with the vowel gesture proper (top panel) and movement that is associated with a sequence of neutral attractor followed by a vowel gesture.

Notably, the neutral attractor analysis does not necessarily require the type of state-feedback discussed for the “downstream target” alternative. In this sense, the neutral attractor account of our data is parsimonious with the two level feedforward model of AP. However, the need for bidirectional interaction between inter-gestural and inter-articulator levels has been argued for elsewhere (Saltzman et al., 1998) and other more recent developments in the AP framework may render neutral attractors less necessary than in earlier work. For example, Nam (2007) pursues the hypothesis that the movement toward and away from constrictions are controlled by independent gestures. On this account, the “split-gesture” hypothesis, it is less clear that a neutral attractor is needed at all to return articulators to a neutral position, as this could be accomplished by the release gesture associated with consonants. Other empirical work has identified cases of anticipatory movements in speech which at times pre-empt the linguistically specified timing pattern and cannot easily be explained by a neutral attractor (Davis et al., 2015; Tilsen et al., 2016). Using real-time MRI, Tilsen et al. (2016) observed a range of idiosyncratic (across speaker) patterns of anticipatory movement during silence. He suggested that neutral attractors, if they were to account for the data, would have to be sensitive to upcoming gestures. Other relevant anticipatory movement phenomena include Whalen (1990), who found that, when reading aloud, speakers plan coarticulation based upon available information in the visual stimulus. Similarly, Davis et al. (2015) observed anticipatory articulatory movements in response to subliminal presentation of words in a masked priming task. These findings suggest that orthographic stimuli, even when brief (<50 ms) or absent until speech initiation, condition anticipatory speech movements. Phonetically sensitive neutral attractors have been suggested elsewhere in the literature (Ramanarayanan et al., 2013) but this proposal would have to be developed significantly to encompass the broader range of articulatory phenomena. Thus, while, in the case of our data, a “standard” neutral attractor, i.e., per Saltzman and Munhall (1989), may be sufficient to account for anticipatory movement, alternative mechanisms, e.g., release gestures, planning gestures or otherwise, “phonetically sensitive” attractors are theoretical developments that could potentially subsume the neutral attractor analysis.

In closing this section, we would like to highlight that the two possible theoretical explanations that we’ve offered for the effect of spatial position on relative timing are not mutually exclusive. The neutral attractor could explain some of the early vowel movements, even if the downstream target hypothesis is also correct. The preceding discussion of neutral attractors notwithstanding, it’s possible that both mechanisms are independently necessary. The relative variability of movement onsets in contrast to movement targets has been noted in past work (Perkell and Matties, 1992) and discussed as evidence against a system of speech timing control driven by movement onsets (Turk and Shattuck-Hufnagel, 2014). While the neutral attractor may explain some of the variability found generally for gesture onsets in this and other studies, we note that the neutral attractor hypothesis does not predict the correlation between consonant (closing phase) duration and C-V lag, which was found to be quite strong. This correlation (C-closing and C-V lag) could instead be attributable to yet another factor, such as a general slowdown (scaling) of the clock related to, e.g., speech rate, or to the interaction between general slowdown and an amplitude-gesture duration tradeoff predicted by non-linear dynamical system. However, such a factor will also predicts a positive correlation between C-V lag and vowel duration, which was not shown in our data (see section “Neutral Attractors”).

### Additional Theoretical Implications

On average, C-V lag (V_onset_ to C_onset_) is positive in our data, which may be driven by the interaction between competing forces on coordination, as per the coupled oscillator model of gesture coordination (Goldstein and Pouplier, 2014). Such positive C-V lag in tone languages has been explained by the hypothesis that the onset of the tone gesture is temporally aligned with the offset of the consonant gesture (anti-phase timing) while the vowel onset is competitively coupled to both the consonant and tone gestures (Gao, 2008). However, if the downstream target hypothesis generalizes to tone, then the positive C-V lag found generally for syllables with lexical tone may also have an alternative explanation in terms of downstream targets. Tones, just as vowels, may be timed with reference to a tonal target or to other downstream landmarks, as opposed to the tone onset. Cross-linguistically, it seems necessary for tones to have different modes of syllable-internal alignment. In Dzongka, for example, tones appear to be left-aligned within the syllable, in that the high and low tones are most distinct near the onset of voicing (Lee and Kawahara, 2018). Tones in Mandarin, in contrast, are differentiated later in the syllable (Moore and Jongman, 1997; Shaw et al., 2013). In Dinka, the timing of tones within a syllable is minimally contrastive (Remijsen, 2013). These cross-linguistic patterns suggest a richer ontology of syllable-internal timing patterns than may be possible if coordination makes reference only to gesture onsets.

## Conclusion

Consonant and vowel gestures in Mandarin were generally not synchronous in our data. The vowel movement typically began after the consonant, which is consistent with past work on Mandarin and other lexical tone languages (Gao, 2009; Hu, 2016; Karlin, 2018; Zhang et al., 2019). The spatial position of the tongue influenced when the vowel movement begins relative to the consonant. This is to our knowledge the first direct evidence that the spatial position of the articulators conditions the relative timing of speech movements in unperturbed speech (c.f., Saltzman et al., 1998). On the face of it, this finding seems to challenge strictly feed-forward models of timing control adding to past experimental evidence for bidirectional interaction between the inter-gestural level and the inter-articulator level of speech movement control. We discussed two possible explanations for the effect. The first proposal involves downstream targets. Movement onsets vary with spatial position to achieve coordination of later articulatory events. In this case, it would be necessary for state-based feedback to inform relative timing. Moreover, since the onset of vowel movement often occurred before phonation (during silence), the relevant state-based feedback must be somatosensory (likely proprioceptive) in nature. The “downstream targets” proposal made some additional testable predictions that are consistent with the data. As consonant duration varies, C-V lag covaries in the manner predicted by an alignment of the vowel target to some landmark in the release phase of the consonant. We also found a correlation between gesture amplitude and the duration of the opening movement of vowels, which is predicted by a non-linear dynamical model of gestures (Sorensen and Gafos, 2016). The second proposal involves neutral attractors which drive articulators toward rest position when they are not under active control of a gesture. This is in many ways a simpler solution in that it treats the effect of spatial position on C-V timing as an epiphenomenon of natural speech preparation. While these are both possible accounts of our data, we note that they are not mutually exclusive and that future research is needed to fully evaluate the proposals. Regardless of the proper theoretical account of this finding, future empirical work investigating the relative timing of movement onsets should factor spatial position into the analysis.

## Data Availability Statement

All datasets generated for this study are included in the article/supplementary material.

## Ethics Statement

This study was carried out in accordance with the recommendations of the Western Sydney University Interval Review Board with written informed consent from all subjects. All subjects gave written informed consent in accordance with the Declaration of Helsinki. The protocol was approved by the Western Sydney University Interval Review Board.

## Author Contributions

JS and W-RC designed the experiment, collected the data, and discussed each stage of the analysis. JS conducted the statistical analysis and wrote the first draft of the manuscript. W-RC made some of the figures. JS and W-RC contributed to the manuscript revision, read, and approved the submitted version.

## Conflict of Interest

The authors declare that the research was conducted in the absence of any commercial or financial relationships that could be construed as a potential conflict of interest.
